# Royal Free Hospital Festival Dinner

**Published:** 1906-12-15

**Authors:** 


### ROYAL FREE HOSPITAL FESTIVAL DINNER.

### A GRATIFYING SUCCESS.

The Duke of Connaught presided over the thirty-fifth Festival Dinner of the above hospital, which was held at the Hôtel Cecil on Wednesday (28th November). Amongst those present were : Lieut.-General Sir Gordon Pritchard, Sir Edwin Durning-Lawrence, Sir R. Melvill Beachcroft and Lady Beachcroft, Sir Clifton and Lady Robinson, Mr. Francis Layland-Barratt, M.P., Capt. H. M. and Mrs. Jessel, Dr. A. G. Phear, Dr. H. Sainsbury, Mrs. Scharlieb, Mr. Charles Burt (Treasurer), and Mr. Charles W. Blackman (Chairman of the Weekly Board). The Royal Army Medical Corps Volunteers (London Companies), under Lieut. Herbert C. Phillips, provided the Guard of Honour to the Duke of Connaught.

H.R.H. the Duke of Connaught, in proposing "Prosperity to the Royal Free Hospital," said that it had been his good fortune on many occasions to plead for the cause of our hospitals—they were many, they were, alas! necessary, and they were excellent. What in particular, he might be asked, could he say to recommend the Royal Free Hospital? There were many points which were almost exceptional. The Royal Free Hospital, which was founded as long ago as 1828, had from its inception admitted free all those who were really deserving of surgical or medical help, or who were too poor to help themselves. That was a very splendid idea. No doubt an idea which it had been very difficult to carry out, but it had been carried out. (Applause.) During the time the hospital had been in existence it had treated 3,000,000 people. The expenses of the hospital were about £15,000 a year, and its certain income only about £5,000. Therefore, he left it to his hearers to imagine what work, what sympathy, what interest must be necessary to carry on this most excellent work. The hospital had 178 beds, the number of in-patients annually received was about 2,500, and the out-patients about 30,000. Another feature of the hospital, one which they must all respect, was that it was the first hospital to open its doors to women practitioners. The result had been most successful. A very large number of ladies had come forward and had received most excellent instruction both in the in- and out-patient wards. The lady doctors had done most excellent work in the colonies, and especially in India. During the years he had served in India he had on many occasions met lady doctors who, he could assure his hearers, by their kindly and Christian feeling to the Indian people, and their medical knowledge had been of the greatest advantage in that country, and had done more to attach it to our own than anything he knew. (Applause.) He had heard from many people that amongst the numerous hospitals of this country there was none that was better and more economically managed, and had produced greater efficiency than the Royal Free Hospital. (Applause.) He said that on good authority—he had asked many people, and they had all told him that they had the highest respect for the very splendid and admirable work of the hospital from its very infancy. (Applause.) The hospital, like every other, had had to make great additions. But there were greater still which required to be done, for instance, the out-patients' department and the operating theatre. Would not all who heard him help to enable that hospital to carry on its good work, and by giving liberally assist it to fulfil the purpose for which it was originally intended. He could assure them that there was no object that was more worthy of their sympathy and assistance than the Royal Free Hospital. (Loud applause.)

Mr. Charles Burt, in response, appealed for annual subscribers, and announced that £7,424 had been received as the result of the Festival Dinner.

Dr. Jno. Walter Carr, in his reply to the toast of "The Medical Staff," said he was only too conscious of the many and urgent needs of the hospital, and, as a member of the Board of Management, equally conscious how difficult it is for even the daily requirements of the hospital to be met, and how almost impossible it is to undertake those large schemes of improvement and extension which are so greatly needed. It is unfortunate, but apparently inevitable, that the expenses of a hospital should constantly increase. The medical staff are sometimes held responsible for this, but they are bound to do all they can to ensure the adoption of the latest methods of diagnosis and treatment for the benefit of the patients, and he fully endorsed all that the Chairman of the Board had stated with regard to requirements for massage, radiography, light treatment, special skin baths, and so on. If ever they ceased to ask for further developments and improvements in the hospital, depend upon it they would have become effete, and the time had arrived when they should be dismissed, and a new, more active, and more progressive staff obtained. The medical staff were anxious to do all they can to reduce expenditure. In 1875 the Alcohol Bill of the hospital amounted to £372, and last year to only £55, a very substantial saving, for which the staff deserve credit. They may fairly claim that for increased expenditure they have secured increased efficiency, for in 1885 the average stay of each patient in the hospital was 26 days, and last year this was reduced to 18½ days. Although the Royal Free is considerably smaller than many of the London General Hospitals, it is the desire of the staff that in no other respect whatsoever, whether in regard to efficiency or equipment, should it be in the least behind the largest and best of these hospitals. He hoped that they would agree with him that the staff would be false to the position in which the Governors have placed them, recreant to their duty, and untrue to the highest traditions of their profession were they to aim at any lower standard or to strive after any inferior ideal. But whilst its first duty is the treatment of sick people, the Royal Free Hospital discharges two other most important functions. It is a training school for nurses, and during recent years, largely owing to the wise guidance of the President of the Hospital, H.R.H. Princess Christian, it has endeavoured by adding to the comfort of the nurses and by improving their surroundings, to attract a better class of women; and to increase the thoroughness of their training.

### The Training of Medical Women.

Each year from fifteen to thirty fully qualified medical women pass from this hospital. An increasing number find work in this country; to an ever-growing extent they get positions in connection with infirmaries, fever hospitals, children's hospitals, asylums, and under the education authorities. Only a year or two ago when the London County Council appointed twenty medical officers to inspect the children in their schools, they deliberately chose no less than six women, all of whom had received their training in the Royal Free Hospital; a striking testimony to the increasing confidence shown in medical women by popularly elected representatives. Others of our medical women have heard the call of the East; they are to be found in the secluded Zenanas of India, in the fœtid streets of Chinese cities, in the decaying towns of Persia, and in many other parts of the Eastern hemisphere. It is difficult, indeed, to estimate the value and importance of the work which they do. He sometimes thought that the hospital actually does even more good indirectly by the services which it renders to humanity through the medical women whom it trains, than it does directly by its treatment of the sick poor whom it receives within its walls. We add up our out-patients and in-patients, we calculate, in some rough sort of way, the number of people whom we restore to health and to the battle of life, but who can number or estimate, who can weigh or calculate in any way whatsoever, the good which is done by medical women, scattered over the face of the globe, by bringing to peoples of varying nationalities the benefits of the training, the knowledge and the skill which they have acquired in the Royal Free Hospital? This, indeed, is no local work, it is not even national work, it is an imperial and cosmopolitan work. Moreover it is a work which goes almost unrewarded, it is but little noticed or honoured, and yet it is work attended by the greatest risks and dangers. Medical women have taken their share in the horrors of the Boxer siege of the Pekin Legations, they have helped in fighting the plague in India, they have dealt with cholera epidemics in various parts of the East; and some of those who have been the ablest and most promising of our students have fallen in the contest. For to-day many a lonely grave in many a distant foreign land bears silent, but eloquent, testimony to the reality of the dangers and the greatness of the perils encountered, without fear and without hesitation, by medical women, who go forth oft times as pioneers of civilisation and as pioneers of Christianity. He appealed therefore, as a member of the medical staff of the hospital and as a teacher in the school, on behalf of this three-fold work. For the work which the hospital does to the utmost of its power and resources in dealing with sickness and suffering in a poor and crowded quarter of London; for the work of training skilled nurses, who leave the hospital to be a help and a blessing to all classes of society; for the work of training medical women, who go forth into the dark quarters of the earth, in this and other lands, there to maintain the high reputation of British women, there to uphold and, if possible, to enhance the best and noblest traditions of British medicine.

Other toasts followed.

